# Explainable machine learning-based mortality risk stratification for older adults with COVID-19: pinpointing core immunological biomarkers and revealing dose-threshold effects

**DOI:** 10.3389/fimmu.2026.1789048

**Published:** 2026-05-25

**Authors:** Lin Luo, Lin Wang, Hao Wang, Hui Li, Ting Liu, Sha Yu

**Affiliations:** 1 Clinical Laboratory, Second Affiliated Hospital of Dalian Medical University, Dalian, China; 2 School of Statistics, Dongbei University of Finance and Economics, Dalian, Liaoning, China

**Keywords:** core biomarkers, COVID-19, machine learning, older adults, prediction model

## Abstract

**Background:**

Older adults are the high-risk group for COVID-19-related death. This study aimed to develop an accurate, efficient, clinically interpretable machine learning (ML) model for predicting mortality risk in this population, using only routine hematological indicators at admission to avoid extra medical costs and radiation exposure.

**Methods:**

2393 COVID-19 patients were enrolled in this retrospective study. Missing values were imputed via Random Forest. RandomOverSampler was utilized during model training to alleviate moderate class imbalance. Feature selection was conducted following the maximum relevance-minimum redundancy principle. Five ML algorithms—Logistic Regression (LR), Support Vector Machine (SVM), Random Forest (RF), XGBoost (XGB), Light Gradient Boosting Machine (LGBM) were optimized via bayesian optimization (BO). We performed 10 rounds of random stratified data splitting; models were fitted on the training set, with intermediate screening and hyperparameter optimization implemented on the validation set. The independent held-out test set was strictly reserved for final performance evaluation. Model performances were assessed using the ROC curve, accuracy, precision, recall, F1-score and brier score. Calibration curve evaluated concordance between predicted probabilities and actual outcomes, and decision curve analysis (DCA) quantified net clinical benefit in clinical practice. Shapley additive exPlanations (SHAP) values and partial dependence plots (PDPs) interpreted feature importance and their associations with mortality risk. A simplified model was further developed using the top 10 key features identified by SHAP analysis and a corresponding risk prediction system was constructed to facilitate clinical application by physicians.

**Results:**

The LGBM model achieved the best comprehensive performance: AUC of 0.973, recall of 0.924, accuracy of 0.918, F1-score of 0.918, NPV of 0.923, and Brier score of 0.064. It outperformed other algorithms in computational efficiency and cross-dataset stability. Top 10 key features identified included basophil percentage (BA%), C-reactive protein (CRP), procalcitonin (PCT), D-dimer (D-dimer), AST/ALT ratio, cardiac troponin I (cTnI), standard bicarbonate (SB), age, aspartate transaminase (AST), and oxygen saturation (SaO_2_) for predicting mortality risk in older adults. Non-linear associations and threshold effects were observed (e.g., risk surged when CRP > 100 mg/L or D-dimer > 5–10 μg/mL). The simplified model reduced training time by 58.31% without compromising performance, comparable accuracy and interpretability.

**Conclusion:**

This study developed a TPE-LGBM model based on routine hematological indicators to predict mortality risk in older adults with COVID-19. The model demonstrated favorable accuracy, efficiency, and interpretability, suggesting the potential value of applying explainable machine learning to address unmet medical needs.

## 1 Introduction

Since the identification of severe acute respiratory syndrome coronavirus 2 (SARS-CoV-2) in late 2019, its associated disease—coronavirus disease 2019 (COVID-19)—has evolved into a global-scale pandemic, exerting an overwhelming strain on healthcare systems worldwide (1). COVID-19 is defined by a short incubation period, high transmissibility, and rapid spread—attributes tightly linked to SARS-CoV-2’s robust immune evasion capacity and its continuous generation of novel variants via immune evasion mechanisms. While the pandemic’s peak has subsided, emerging variants (e.g., JN.1) are undergoing accelerated global transmission, posing a persistent risk of epidemic resurgence (2). Strikingly, COVID-19 patients exhibit marked heterogeneity in clinical manifestations and prognosis: those with mild disease typically clear infection via early innate immunity, whereas patients with severe illness or immunocompromised individuals (e.g., older adults, those with chronic comorbidities, and malignancy patients) are predisposed to progress to multiorgan dysfunction, intensive care unit (ICU) admission, or mortality (3). With the global elderly population expanding and chronic disease prevalence rising, the cohort at high risk of severe COVID-19 has further enlarged (4). Compounding this, COVID-19 shares symptom overlap and comparable transmission routes with seasonal influenza; during influenza outbreaks, it is frequently misidentified or overlooked, leading to treatment delays that contribute to rapid progression from mild initial symptoms to severe illness. Accordingly, accurate and timely mortality risk stratification for COVID-19 patients is paramount to optimizing clinical management strategies and the rational allocation of limited healthcare resources. Clinically, there remains an unmet need for accessible point-of-care early warning tools to identify patients at high risk of poor prognosis. Such tools would enable proactive interventions to ameliorate clinical outcomes, reduce hospitalization and mortality risks, and prioritize resource allocation to those with the greatest clinical need (5)—ultimately enhancing the overall COVID-19 patients management and lowering mortality.

With the rapid advancement of computational technology, machine learning (ML) has been widely deployed in developing clinical prediction models for diverse diseases. Centered on data mining algorithms and predictive analytics, ML facilitates large-scale analysis of multi-dimensional data—including patient vital signs and laboratory results—to precisely identify individual disease phenotypes and subtle pathological changes. Beyond capturing non-linear associations in datasets and overcoming limitations of traditional statistical modeling (e.g., logistic regression), it has demonstrated superior predictive performance (6, 7); in select scenarios, it has even exhibited more efficient disease assessment capabilities than clinicians (8–11). Leveraging its high predictive accuracy, ML models support clinicians in promptly formulating evidence-based clinical decisions and delivering targeted interventions during early disease stages, thereby mitigating the risk of adverse outcomes and providing critical backing for clinical management.

In the COVID-19 field, early prediction models relied heavily on traditional scoring systems (12, 13). While recent studies have attempted to construct ML-based models, most have focused on the general population, with no targeted investigations addressing older adults at high risk of severe illness (14, 15). Notably, the older adults bear the highest COVID-19 mortality risk and thus demand prioritized attention: studies confirm that older adults, due to immunosenescence and chronic inflammatory states, are more susceptible to progressing to severe COVID-19 (10, 11).Data from the U.S. Centers for Disease Control and Prevention (CDC) show over 81% of COVID-19 deaths occurred in individuals aged ≥65 years—with a mortality rate 97-fold higher than that of adults aged 18–29 years (16). Additionally, older adults present with insidious onset and high rates of delayed diagnosis (diagnosis latency >24h); this delay is significantly associated with elevated severe illness risk, further exacerbating adverse outcome likelihood (17). Emerging evidence confirms that early interventions for populations at high risk of severe COVID-19 substantially reduce their hospitalization and all-cause mortality rates (18, 19). Accordingly, developing early risk screening tools for this cohort holds considerable clinical value.

Herein, we exclusively used routine hematological indices obtained at patient admission (no additional tests required, thereby avoiding unnecessary ionizing radiation exposure and mitigating additional testing burden and economic costs for patients). By constructing multiple ML algorithm-based models, we ultimately developed a COVID-19 mortality risk prediction model. This study aims to enable precise assessment of COVID-19 patients’ mortality risk via this model, providing evidence-based support for clinicians to refine patient stratification and management strategies and reduce mortality.

## 2 Methods

### 2.1 Study design and setting

This retrospective cohort study was designed and conducted in accordance with the Strengthening the Reporting of Observational Studies in Epidemiology (STROBE)statement. Data were retrieved from patients admitted to the Second Affiliated Hospital of Dalian Medical University for COVID-19 treatment between December 1, 2022, and August 31, 2025.

### 2.2 Patient selection and cohort derivation

**Figure 1:**
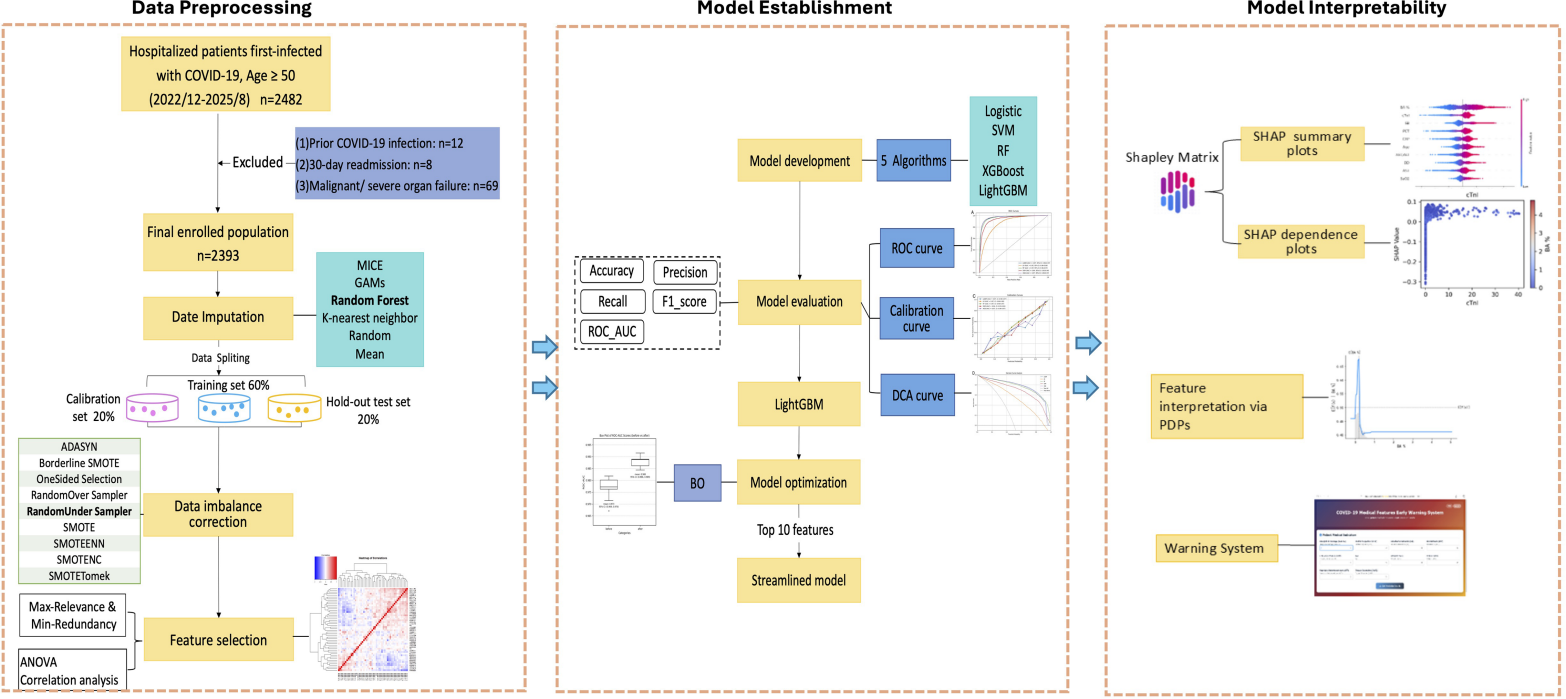
The flowchart of patient screening, eligibility assessment and the overall study workflow. The model was fitted on the training set; intermediate model selection and hyperparameter optimization were performed on the validation set, with probability calibration implemented where necessary. Final performance was evaluated exclusively on the corresponding independent held-out test set.

To ensure the representativeness of the study population and minimize selection bias, patients were included consecutively based on their admission date. Eligibility criteria for study enrollment were strictly defined as follows: (1) age ≥ 50 years; (2) confirmed as first-time SARS-CoV-2 infection via real-time reverse transcription-polymerase chain reaction (RT-PCR) testing of nasal or pharyngeal swab samples. Patient-level exclusions were applied after initial screening as follows: (1) Patients with a documented history of prior SARS-CoV-2 infection. (2) Readmissions within 30 days of discharge for the same infection episode (to avoid duplicate counting of the same clinical episode). (3) Patients with concurrent malignant tumors or severe organ failure. The primary endpoint of the study was defined as in-hospital mortality. The final study cohort was derived by applying these criteria to the total eligible population. **Figure 1** provides a detailed flowchart illustrating patient screening, eligibility assessment and the overall study workflow.

The age cutoff of ≥50 years was selected as an inclusion criterion based on multiple lines of evidence. First, epidemiological studies have consistently demonstrated that age is a key predictor of COVID-19 mortality, with a sharp increase in mortality risk observed from 50 years of age in the Chinese population (20). Second, the ≥50 years threshold is consistent with international vaccination priority guidelines: the U.S. Advisory Committee on Immunization Practices (ACIP) lowered the age recommendation for annual influenza vaccination from 65 to 50 years as early as the 2000–2001 season (21), Nordic countries and Switzerland have restricted COVID-19 booster vaccine eligibility to individuals aged ≥50 years and vulnerable populations (22). Third, this cutoff aligns with risk stratification criteria used in numerous domestic and international studies. The Robert Koch Institute (RKI) in Germany designates 50–60 years as a range with steadily increasing severe disease risk (23), and similar cutoffs have been adopted in Korean and Japanese studies focusing on severe illness and mortality outcomes (24, 25). Finally, Immunosenescence and the decline in multi-organ functional reserve typically begin to manifest around the age of 50, making this threshold appropriate for early identification of high-risk individuals and precise risk stratification in the Chinese population.

### 2.3 Data collection and laboratory measures

Patient-related information—including demographic characteristics and routine laboratory test results—was extracted from the hospital’s Laboratory Information System (LIS) and Electronic Medical Records (EMR). To capture the baseline physiological status and minimize the influence of therapeutic interventions, we selected the first available laboratory measurements obtained within the first 24 hours after hospital admission as the index values. Although multiple laboratory tests were recorded during the hospitalization, only the baseline (admission) values were utilized for the primary statistical analysis to ensure a consistent exposure definition across all participants. Sensitivity analyses regarding the impact of time-varying covariates were not performed for this primary report.

### 2.4 Bias control

To mitigate potential sources of bias, we implemented the following strategies: First, we attempted to lessen selection bias by consecutively enrolling all eligible hospitalized patients during the study period, thereby limiting selective recruitment related to clinical outcomes. Second, information bias was partially mitigated by directly extracting laboratory data from the LIS, which minimized manual transcription errors. We compared and evaluated multiple missing value imputation methods and ultimately adopted random forest imputation due to its lowest mean squared error. Third, confounding bias was partially mitigated by the standardized clinical management provided to all patients. During the study period, all participants were treated in accordance with a unified local COVID-19 protocol, with no substantial differences in therapeutic strategies, supportive care, or resource allocation. This standardized approach helped lessen potential confounding arising from differential treatment pathways. Fourth, to address time-related bias, exposure indicators were strictly restricted to data collected within the first 24 hours of admission, and clinical outcomes were assessed based on the entire clinical course. This approach helped limit the influence of immortal time bias.

### 2.5 Ethics statement

This retrospective study was reviewed and approved by the Ethics Committee of the Second Affiliated Hospital of Dalian Medical University (Ethical Approval Number: KY2025-537-01). The requirement for informed consent was waived by the Ethics Committee of the Second Affiliated Hospital of Dalian Medical University because this retrospective observational study only utilizing routinely collected clinical data without any experimental intervention. Given that the study analyzed anonymized historical data and contacting all patients was not feasible, the committee determined that this study met the criteria for a waiver of informed consent in accordance with the Measures for Ethical Review of Biomedical Research Involving Humans and the principles of the Declaration of Helsinki. All patient data were de-identified to protect privacy, and no personally identifiable information was disclosed in the manuscript.

### 2.6 Addressing missing values

In clinical data collection, missing values are pervasive, introducing bias and compromising analytical reliability. Naive deletion of records containing missing values risks losing valuable information and exacerbating bias (26). From the original 197 features, we adopted a conservative exclusion strategy: variables with a missing rate exceeding 90% were deemed invalid and excluded, reducing the feature space to 128 variables. The overall missing rate in the remaining dataset was 21.30%. Missing data in clinical datasets often exhibit “structural missingness,” where specific laboratory tests are ordered based on clinical indications (e.g., glucose testing is often absent in non-diabetic patients). To ensure the robustness of our imputation, we employed a simulated missingness validation approach to select the optimal method.

The detailed implementation strategy is presented below: we randomly selected 1000 samples from a relatively complete data subset and artificially introduced missing values at two gradients (10% and 20%) to mimic real-world patterns. Subsequently, six imputation methods were evaluated, including: random imputation (based on random sampling from clinical reference ranges); mean imputation (utilizing median for ordinal, mode for categorical); K-nearest neighbor imputation (imputing via the mean of the most similar samples in feature space); generalized additive models imputation (GAMs, predicting values for imputation via constructing additive nonlinear smoothers); random forests imputation (constructing multiple decision trees and aggregating results via voting/averaging); and multiple imputation by chained equations (MICE, which generates and integrates multiple complete datasets via chained equations). Imputation accuracy was quantified using the mean squared error (MSE) over 10 repeated simulations. The simulation results demonstrated that random forest (RF) imputation consistently achieved the lowest MSE and highest accuracy across all variables, outperforming both the rule-based random imputation and other algorithmic methods (**Table 1**). Consequently, the RF method was selected as the single, unified imputation technique in the final dataset due to its ability to model non-linear relationships and handle high-dimensional, skewed distributions without requiring assumptions of normality. While RF imputation can effectively estimate missing values based on observed data patterns, we acknowledge that it may not fully reflect the true clinical status behind missing laboratory tests, especially for variables with prominent missing data, where imputation fails to fully capture inherent clinical information. The final dataset included 2393 patients with 128 features (see **Supplementary Table 1** in **Supplementary Material 1** for details).

**Table 1:** Comparison of model prediction performance under different imputation strategies.

Metric	Missing	Random imputation	Mean imputation	KNN imputation	GAM imputation	RF imputation	MICE imputation
MSE	10%	1469.867	1253.320	2.981	975.071	0.067	89.929
	20%	2034.279	1714.58	560.008	1452.968	0.417	340.555

### 2.7 Addressing imbalanced data

Moderate class imbalance was observed for the study’s endpoint (mortality): 84.45% survival, 15.55% mortality, and the survival-to-mortality ratio was 5.43:1. To address this, nine balancing techniques were applied: seven oversampling methods [Synthetic Minority Oversampling Technique (SMOTE), Adaptive Synthetic Sampling (ADASYN), SMOTE with Tomek Links (SMOTETomek), SMOTE with Edited Nearest Neighbors (SMOTEENN), BorderlineSMOTE, SMOTENC, RandomOverSampler] and two undersampling methods [RandomUnderSampler, One-Sided Selection]. By comparing performance metrics (accuracy, precision, recall, F1-score, ROC-AUC) of models trained on technique-processed data, RandomOverSampler was ultimately selected as optimal strategy. Notably, the RandomOverSampler algorithm was exclusively applied during model training to mitigate class imbalance. The performance parameters of these balancing technologies were shown in **Table 2**.

**Table 2:** Performance metrics of models constructed with data processed by different techniques.

Data processing methods	MLs	Accuracy	Precision	Recall	F1_score	Roc_auc
Raw Data	LGBM	0.8668	0.6249	0.3639	0.4583	0.8597
	LR	0.8678	0.6422	0.3542	0.4554	0.8473
	RF	0.8676	0.6843	0.2827	0.3993	0.8539
	SVM	0.8608	0.7068	0.1849	0.2924	0.8440
	XGB	0.8676	0.6340	0.3620	0.4584	0.8479
ADASYN	LGBM	0.8546	0.4955	0.4544	0.4739	0.8411
BorderlineSMOTE	LGBM	0.8706	0.5606	0.4732	0.5128	0.8358
OneSidedSelection	RF	0.8678	0.5932	0.3167	0.4122	0.8571
RandomOverSampler	LGBM	0.9241	0.9112	0.9397	0.9252	0.9761
RandomUnderSampler	XGB	0.9265	0.9113	0.9448	0.9277	0.9780
SMOTE	LGBM	0.8323	0.4560	0.4440	0.4486	0.8191
SMOTEENN	LGBM	0.8135	0.4478	0.6834	0.5402	0.8379
SMOTENC	RF	0.8671	0.5728	0.5541	0.5602	0.8713
SMOTETomek	RF	0.8566	0.5635	0.5625	0.5618	0.8493

Owing to space limitations, only the best-performing model for each processing strategy was shown when comparing model performance before and after processing. All table values are the mean of 10 repeated tests. Results indicated that the XGBoost model with RandomUnderSampler yielded the highest performance; however, given significant information loss with undersampling, RandomOverSampler (second-best performance, closely approximating the optimal) was selected. RandomOverSampler offers the advantage of preserving the original sample distribution balance and maintaining the inherent distributional characteristics of the data by duplicating existing minority-class samples. For the formal model construction, selection and result validation, a 60% training set:20% calibration set:20% test set stratified random split was strictly adopted.

### 2.8 Feature selection

**Figure 2:**
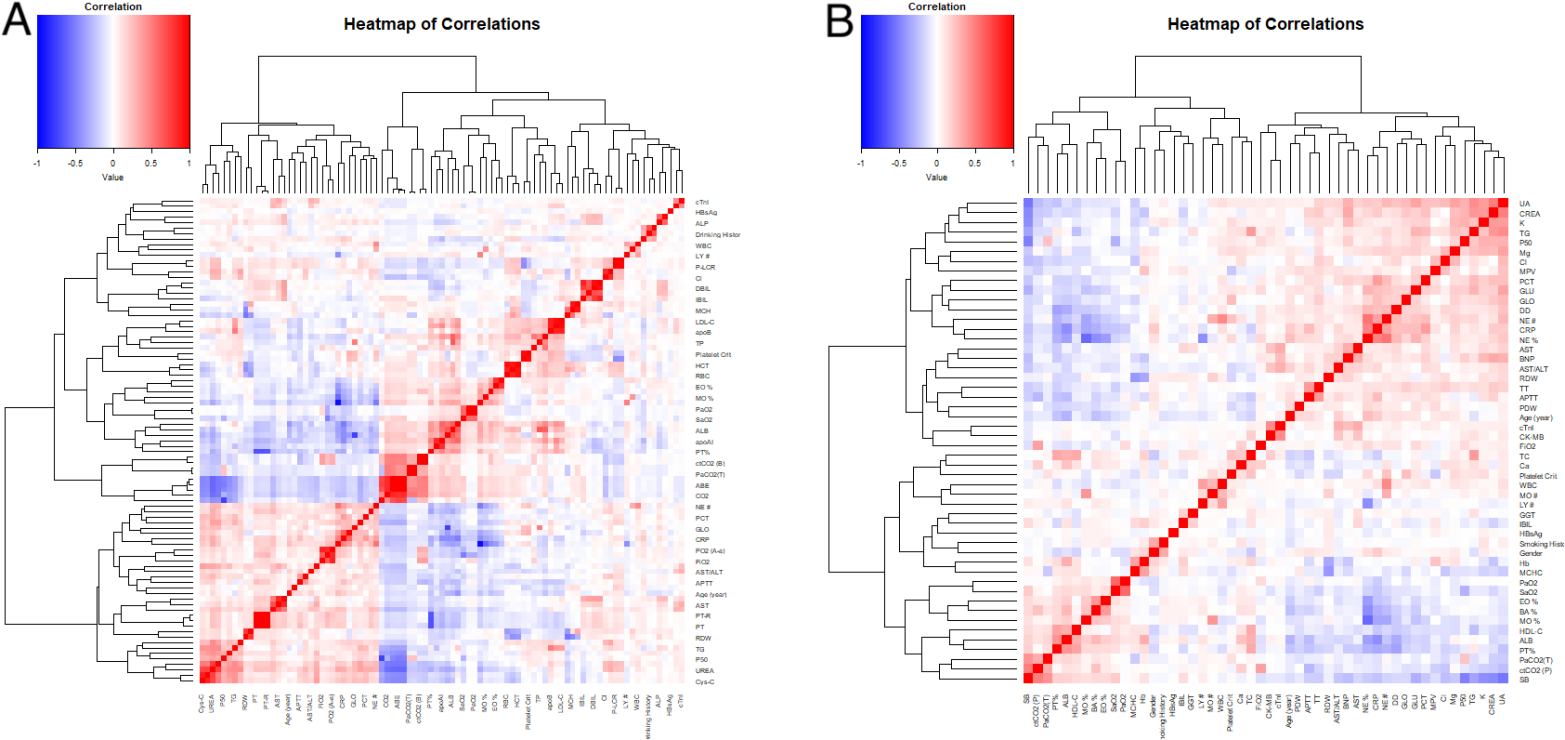
Clustered correlation heatmap [**(A)** raw data, **(B)** screened data]. Red denotes positive correlations, blue negative ones; color intensity reflects correlation strength. Pre-screening, the heatmap showed scattered dark red (strong positive) and dark blue (strong negative) regions, indicating extensive inter-feature redundancy. Post-screening, high-correlation regions decreased markedly, confirming effective removal of redundant features while preserving independent associations without altering intrinsic feature relevance. This validates the strategy retains predictive features, eliminates redundancy, simplifying models and enhancing interpretability. The two correlation heatmaps presented in this manuscript were plotted using the full dataset, serving solely to illustrate the overall changes in the variable correlation structure before and after feature selection.

Given the large number of variables, high redundancy, and strong multicollinearity in the dataset, a filter-based feature selection approach was adopted prior to model training to remove uninformative or redundant features based on statistical tests and correlation structures. Drawing on the Sure Independence Screening (SIS) framework proposed by Zhang (2008), we performed an initial screening of high-dimensional clinical laboratory data according to the marginal correlation between candidate features and the target outcome (27). This step reduced the high-dimensional feature space to a computationally tractable scale, after which features were further selected under the max-relevance and min-redundancy (mRMR) criterion. The mRMR principle prioritizes a subset of features that exhibit the strongest associations with the study endpoint while minimizing redundancy among the selected features, thereby preserving predictive information and reducing instability and interpretive bias caused by multicollinearity. Specifically, the max-relevance component was implemented following the SIS paradigm: one-way analysis of variance (ANOVA) was used to identify features significantly associated with the target outcome (patient mortality), and only those showing significant associations were retained to ensure predictive validity. For the min-redundancy component, pairwise Pearson correlation coefficients were computed among the preselected features. For any pair of features with a correlation coefficient exceeding 0.6, two senior clinicians jointly evaluated the practical clinical value of each feature (e.g., accessibility, relevance to pathophysiological mechanisms), and determined the priority for retention. Only one representative feature was retained for modeling, and the other was excluded. After verification, a total of 50 features were retained in the final test run for subsequent modeling. This approach achieves dimensionality reduction by optimizing feature relevance, lowering feature count while minimizing redundancy with highly correlated clusters. Feature comparison pre- and post-screening is in **Figure 2**.​ To interpret black-box models and quantify the relationships between individual features and COVID-19 mortality, partial dependence plots (PDPs) visualize each feature’s average marginal effect on mortality risk prediction, controlling for other covariates. This captures non-linear relationships and threshold effects often missed by traditional regression.

### 2.9 Model development, optimization, and evaluation

In this study, we developed accurate, robust COVID-19 mortality prediction models using five machine learning algorithms—Logistic Regression (LR), Support Vector Machine (SVM), Random Forest (RF), Extreme Gradient Boosting (XGBoost, XGB), and Light Gradient Boosting Machine (LightGBM, LGBM)—with mortality as the target variable. Within a rigorous machine learning evaluation framework, the training set, calibration set, and held-out test set should serve distinct roles. Specifically, the held-out test set is not involved in model training or calibration and is exclusively used for independent performance evaluation. In each replication, data were randomly split into training, calibration, and held-out test sets at a ratio of 6:2:2. The model was fitted on the training set; intermediate model selection and hyperparameter optimization were performed on the validation set, with probability calibration implemented where necessary. Final performance was evaluated exclusively on the corresponding independent held-out test set. Notably, instead of using a single fixed test set for one-time evaluation, we adopted 10 repetitions of random stratified splitting. This design was intended to reduce variability caused by single random splits and to report the average generalization performance and stability of the model across different data partitions. Accordingly, our results should be interpreted as repeated hold-out internal validation rather than a single final hold-out confirmation. To further improve predictive performance, models were optimized using the Stacking ensemble framework combined with Bayesian Optimization (BO). For BO, model training was performed on the training set, the optimal hyperparameters were selected based on the AUC on the calibration set, and the final model performance was evaluated on the held-out test set. Stacking integrates outputs from multiple base learners and utilizes a meta-learner to train a high-level model for final inference, capturing the complementarities of base learner to reduce bias/variance and enhance generalization. As a Bayesian-theory-driven global optimization method, BO iteratively updates the surrogate model by sequential evaluation points to approximate the objective function’s optimal solution. It outperforms traditional grid/random search in rapidly localizing the optimal solution and reduces computational costs for high-dimensional data.

Model performance was assessed using the receiver operating characteristic (ROC) curve, accuracy, precision, recall, F1-score, area under the ROC curve (AUC-ROC), negative predictive value (NPV) and Brier score. The calibration curve evaluated the concordance between the predicted probabilities and actual observed outcomes, while decision curve analysis (DCA) quantified the net clinical benefit of various models in the context of clinical practice. For interpretability, Shapley Additive exPlanations (SHAP) quantified each feature’s contribution to model outputs, facilitating model interpretation and extraction of key predictive features. Rooted in game theory, SHAP explains model predictions by additive feature attribution, expressing the outputs as linear aggregations of input variables. It fairly allocates variable effects across the full dataset to assess feature contributions and captures inter-feature interactions (28).

### 2.10 Statistical analysis

In descriptive analyses, continuous variables were summarized as the median (interquartile range, IQR), and categorical variables were summarized as frequency (percentage). Missing value imputation was conducted via the R package missForest. Addressing data imbalance was performed using a combination of the R package ‘ROSE’ and the Python package ‘imblearn’. Model training and optimization, incorporating the algorithms LR, SVM, RF, XGB, and LGBM, were implemented via the Python package ‘scikit-learn’. ROC curve generation and AUC calculation were conducted using the R package ‘pROC’; calibration curves and Brier scores were derived using the R package ‘rms’.

## 3 Result

### 3.1 Baseline characteristics of the subjects

A total of 2393 patients were enrolled in this study, including 1397 males (58.38%) and 996 females (41.62%), with a mean age of 74± 11 years. The mortality rate was 15.50% (371 cases). Detailed baseline demographic data and laboratory test indicators are presented in **Supplementary Table 1**.

### 3.2 Feature interpretation via PDPs

**Figure 3:**
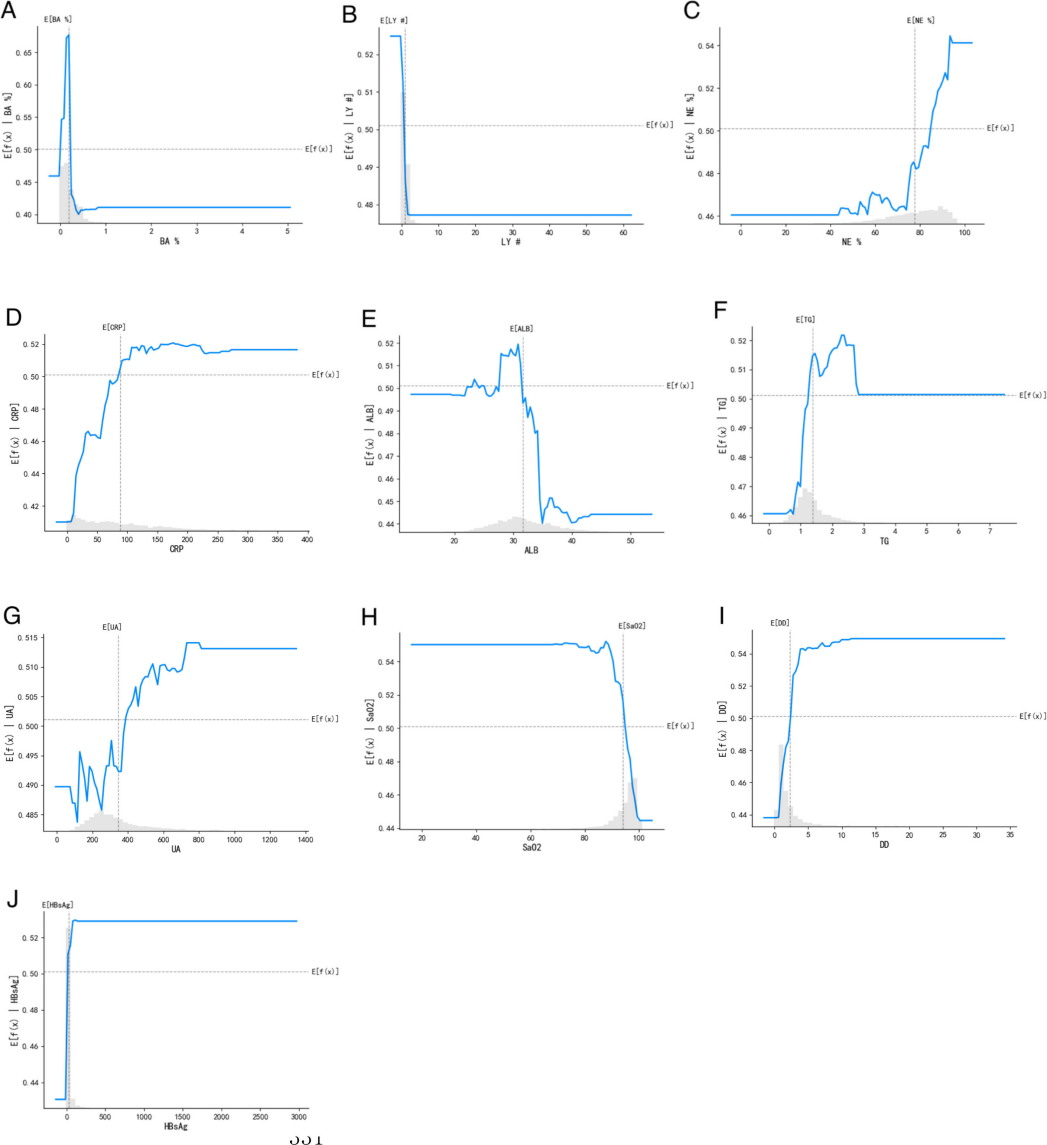
Threshold effects of hematological, biochemical, and oxygenation markers on mortality risk. [**(A)** BA, **(B)** LY#, **(C)** NE%, **(D)** CRP, **(E)** ALB, **(F)** TG, **(G)** UA, **(H)** SaO_2_, **(I)** DD, **(J)** HBsAg].

PDPs revealed prominent nonlinear and threshold-related statistical patterns between multiple laboratory markers and predicted mortality risk. Low levels of basophil percentage (BA%), lymphocyte count (LY#), and oxygen saturation (SaO_2_) were all associated with a marked elevation in predicted mortality risk among older adults, which declined significantly as these indices rose above their respective model-derived threshold values. Conversely, elevated levels of neutrophil percentage (NE%), C-reactive protein (CRP), uric acid (UA), D-dimer (DD), and hepatitis B surface antigen (HBsAg) corresponded to persistently higher predicted mortality risk in the model. Triglycerides (TG) exhibited a nonlinear pattern, with mortality risk increasing rapidly within an intermediate concentration range before reaching a plateau. Albumin (ALB) showed a clear threshold-related favorable prognostic trend, with levels above 35 g/L associated with substantially more favorable predicted outcomes. Collectively, these PDP profiles illustrate that the predictive value of immune, inflammatory, metabolic, and hematological markers for mortality is highly context-dependent and characterized by critical model-identified cutoff values, as detailed in **Figure 3**.

The x-axis represents the feature range and the y-axis shows the partial effect on the predicted outcome. The blue solid line represents the estimated partial dependence function, while the gray shaded region indicates the 95% confidence interval.

- The PDP for BA% shows that BA% approaching 0 was associated with a sharp increase in predicted mortality risk, Once BA% exceeded 0.5%, the predicted mortality risk decreased significantly.
- The PDP for LY# reveals that values approaching 0 are associated with sharply elevated predicted mortality risk, which declines progressively with increasing LY#.
- NE% shows a distinct threshold pattern in its PDP. Values below 80% correspond to relatively low, fluctuating mortality risk, whereas levels exceeding 80% are accompanied by a sharp and sustained rise in predicted risk.
- The PDP for CRP demonstrates a progressive increase in predicted mortality risk with rising concentrations up to 100 mg/L, beyond which risk remains high and variable.
- A clear threshold effect is observed in the PDP for ALB. Levels below 35 g/L are associated with markedly higher mortality risk, while values above this cutoff predict substantially more favorable outcomes.
- The PDP for TG displays that predicted mortality risk is low at levels below 1.0 mmol/L, increases rapidly with rising TG between 1.0–2.5 mmol/L, and plateaus at its highest level within this range.
- The PDP for UA highlights a gradual but persistent increase in mortality risk once levels exceed 400 μmol/L.
- SaO_2_ exhibits a sharp inflection in its PDP. Values below 85% correspond to persistently elevated mortality risk, which decreases notably as SaO_2_ rises above this level.
- The PDP for DD identifies a critical threshold at approximately 5–10 ng/mL. Beyond this point, predicted mortality risk increases abruptly and remains consistently elevated.
- For hepatitis B surface antigen (HBsAg), the PDP shows that risk remains low at near-zero values but increases sharply once detectable levels are present, with persistently high risk across the positive range.

### 3.3 Model development, evaluation and selection

Considering the characteristics of the predictive task, we compared five algorithms: logistic regression (LR), support vector machine (SVM), random forest (RF), XGBoost (XGB), and LightGBM (LGBM). Model predictions were obtained via 10-fold cross-validation, with model performance metrics summarized in **Table 3**. The optimal model was determined through a comprehensive assessment of diverse metrics, including numerical metrics (AUC, accuracy, precision, recall, F1-score, Brier score) and graphical metrics (receiver operating characteristic (ROC) curve, calibration curves, decision curves).

**Table 3:** Comparison of performance metrics among the five models.

Model	Accuracy	Precision	Recall	F1_score	Roc_auc	NPV	Brier_score
LGBM	0.918	0.912	0.924	0.918	0.973	0.923	0.064
LR	0.786	0.776	0.805	0.790	0.867	0.798	0.147
RF	0.908	0.908	0.909	0.908	0.967	0.909	0.069
SVM	0.877	0.868	0.889	0.878	0.939	0.886	0.094
XGB	0.917	0.909	0.928	0.918	0.973	0.926	0.066

**Figure 4:**
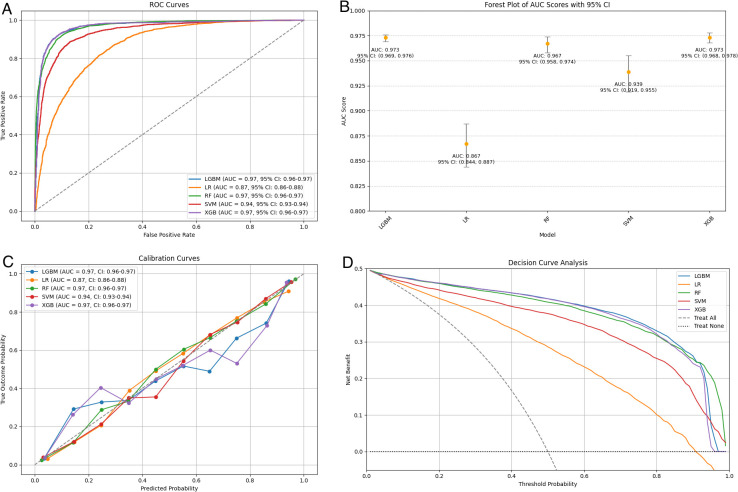
Evaluation of the 5 ML models. **(A)** ROC curves for the five models. **(B)** Forest plot of the AUCs of the five models. **(C)** The calibration curves of the five models. **(D)** The decision curves of the five models.

Both XGB and LGBM exhibited exceptional performance in accuracy metrics. Their ROC curves clustered tightly near the upper-left corner, indicating their superior predictive accuracy and comparable capability in distinguishing between the positive and negative samples, with no statistically significant difference in their core discriminative efficacy (**Figure 4A**). In the forest plot, LGBM yielded an AUC of 0.973 (95% CI: 0.969, 0.976), with a narrower CI than XGBoost, indicating superior predictive stability (**Figure 4B**). Calibration curves demonstrated that the LGBM and XGB predictions aligned closely with actual outcomes along the diagonal, confirming high concordance with real-world results. Notably, LGBM displayed marginally smaller calibration errors in the mid-to-low probability range (0.2–0.6), better aligning with the risk stratification distributions (**Figure 4C**). Decision curve analysis (DCA) revealed that LGBM and XGB yielded similar net benefits across the critical threshold range (0.2–0.8), and both outperformed other models significantly (**Figure 4D**). As shown in **Table 3**, LGBM and XGB exhibited negligible differences in accuracy (0.918 vs. 0.917), precision (0.912 vs. 0.909), and negative predictive value (NPV: 0.923 vs. 0.926). More importantly, cross-validation stability tests confirmed LGBM’s low hyperparameter sensitivity, with small performance fluctuations across the dataset subsets, indicating superior long-term deployment robustness. Additionally, LGBM required 92.83% of XGB’s computational time, and only 11.71% and 12.00% of RF’s and SVM’s respectively, offering a substantial efficiency advantage in real-time prediction scenarios by markedly reducing time costs. LGBM also showed excellent performance in recall and Brier score—two metrics critical for minimizing missed diagnoses and prediction errors. Especially in healthcare and other fields with extremely low tolerance for missed diagnoses, LGBM minimizes false negatives to the greatest extent, ensuring the reliability of results. Based on a comprehensive evaluation of the aforementioned metrics, LGBM was ultimately designated as the optimal model for subsequent investigations.

### 3.4 Model optimization

**Figure 5:**
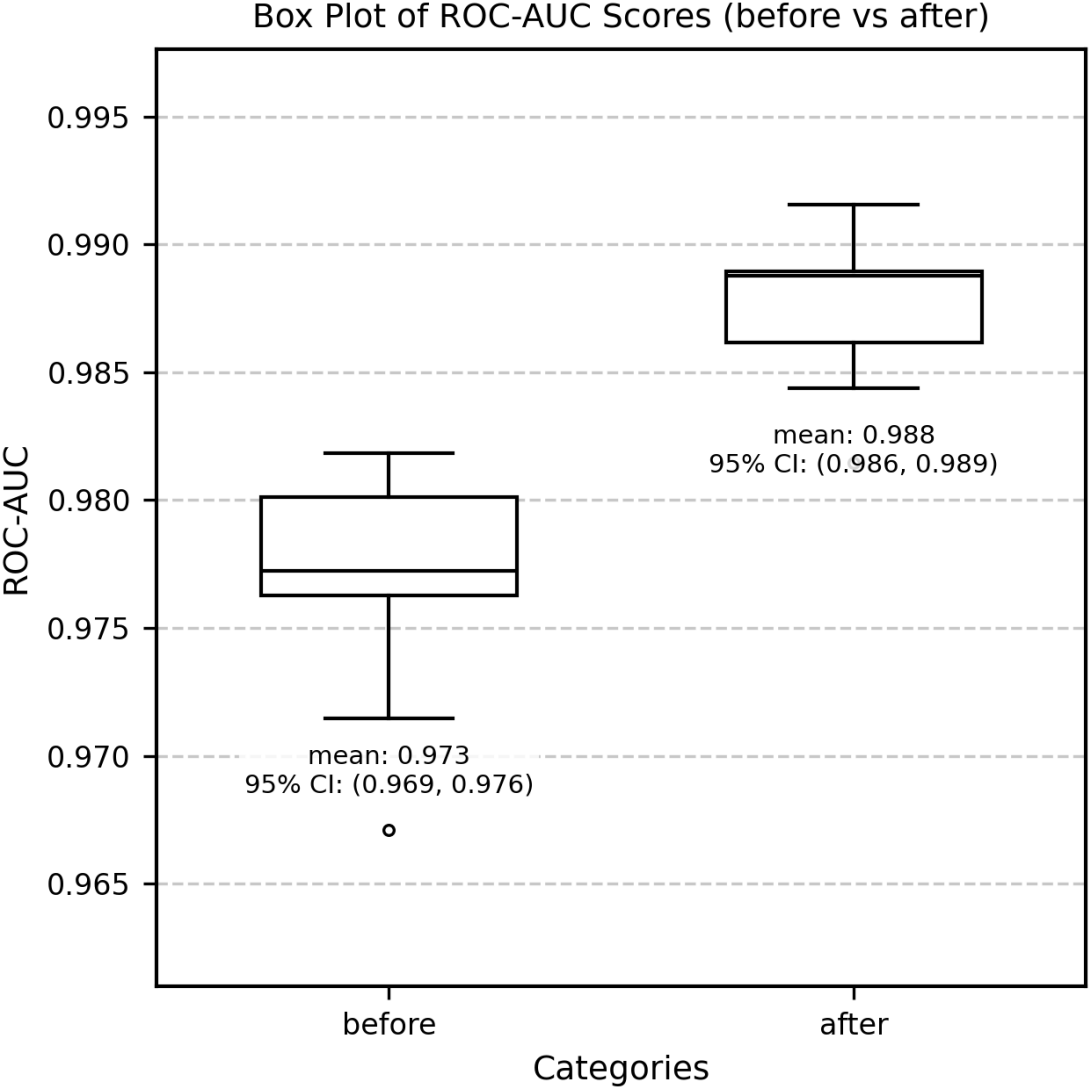
AUC comparison of LightGBM model before and after Bayesian optimization.

Bayesian optimization (BO) based on the tree-structured Parzen estimator (TPE) algorithm was employed to optimize the hyperparameters of the Light Gradient Boosting Machine (LGBM) model. The final optimized model is denoted as TPE–LightGBM. Box plots were employed to facilitate comparative benchmarking of model accuracy, enabling both visual and quantitative efficacy evaluation of the optimization. Following Bayesian optimization (BO)-driven tuning, the mean AUC of the LGBM model significantly improved, reaching 0.988. As visualized in the box plot distribution (**Figure 5**), the median accuracy after optimization exhibited a notable upward shift, accompanied by a modest expansion of the interquartile range. By systematically sampling the hyperparameter space through probabilistic surrogate models, BO identified configurations more commensurate with inherent data patterns, thus enhancing predictive performance.

### 3.5 Development and validation of the streamlined model

To enhance model interpretability, reduce computational complexity and mitigate the risk of overfitting, we conducted SHAP analysis on the finalized hyperparameter-optimized TPE-LGBM model subsequent to completing model selection and parameter tuning. We first calculated the SHAP values of all variables for this model and ranked the features globally by their mean absolute SHAP values, thereby identifying variables with substantial contributions to model predictions. The top 10 ranked features were selected to construct a simplified model, which was then retrained and validated under the identical data partitioning strategy and training workflow applied to the original model. This step was designed to evaluate whether the core feature subset could effectively retain the key predictive information of the original model. As summarized in **Table 4**, the streamlined model demonstrated predictive performance comparable to its full-featured counterpart, while achieving a 58.31% reduction in training time and enhanced interpretability. This outcome validates that the selected feature subset can effectively capture the core predictive information.

**Table 4:** Prediction performance of the streamlined models.

Model	Accuracy	Precision	Recall	F1_score	Roc_auc	NPV	Brier_score
LGBM	0.900	0.892	0.910	0.901	0.960	0.908	0.077
LR	0.772	0.773	0.769	0.771	0.855	0.771	0.155
RF	0.889	0.886	0.892	0.889	0.952	0.892	0.084
SVM	0.806	0.797	0.820	0.809	0.888	0.816	0.135
XGB	0.901	0.892	0.913	0.902	0.962	0.911	0.077

### 3.6 Model interpretability via SHapley additive exPlanations values

**Figure 6:**
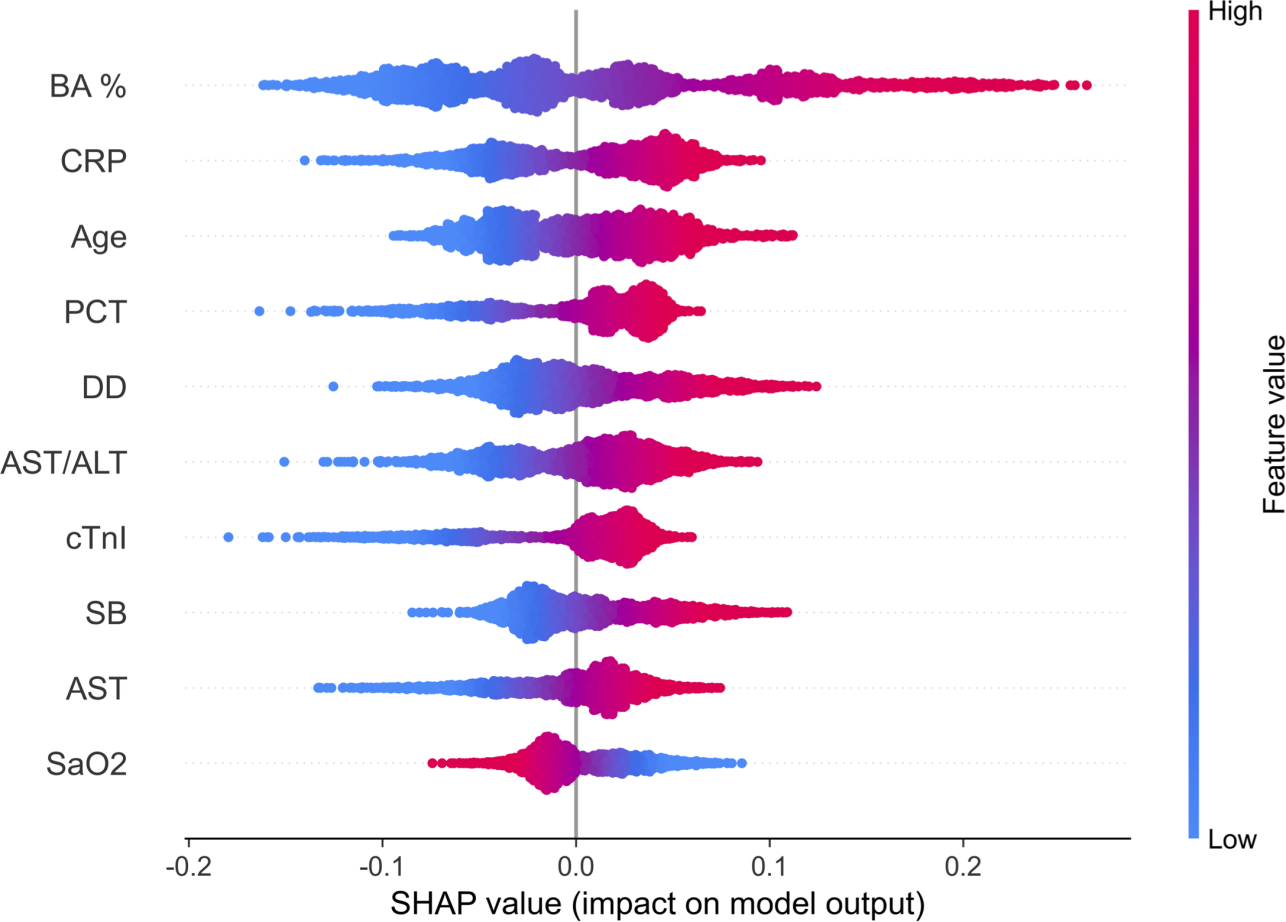
Contribution of the 10 parameters for LightGBM model. The x-axis represents SHAP values, which characterize the direction and magnitude of each feature's impact on model predictions. Specifically, positive SHAP values drive the model output toward an increased risk of COVID-19-related mortality, whereas negative values exert the opposite effect. The distance of a data point from the baseline (SHAP = 0) is proportional to the feature's influence on the model output: the greater the distance, the stronger the impact. Additionally, red indicates high values of the respective feature, while blue corresponds to low values.

To enhance the interpretability of the final model, we performed SHAP analysis on the optimized TPE-LGBM model only after completing model selection and hyperparameter tuning. The hyperparameters of the TPE-LGBM model used for SHAP analysis are as follows: boosting_type = dart, num_leaves = 116, max_depth = 46, learning_rate = 0.386761887, n_estimators = 538, min_child_weight = 5.84973E-05, subsample = 0.428822864, colsample_bytree = 0.359357458, reg_alpha = 0.000189141, reg_lambda = 1.88559E-05. The SHAP summary plot orders features in descending order of mean absolute SHAP values, systematically depicting the global influence of each variable on the model’s predictive architecture. The top 10 features, ranked by their impact magnitude, are sequentially basophil percentage (BA%), C-reactive protein (CRP), age, procalcitonin (PCT), D-dimer (DD), aspartate aminotransferase-to-alanine aminotransferase ratio (AST/ALT), cardiac troponin I (cTnI), standard bicarbonate (SB), aspartate aminotransferase (AST) and oxygen saturation (SaO_2_). For BA%, the feature’s high-value cohort (visualized in red) demonstrates a pronounced rightward localization along the SHAP value distribution, with positive values predominating. This pattern indicates that elevated basophil percentages are positively associated with increased mortality risk in COVID-19 in the predictive model. Conversely, low BA% values (encoded in blue) exhibit a leftward concentration around negative SHAP values, implying a statistically protective correlation with poor prognosis observed in the model. The wide interquartile spread of BA% across the SHAP value spectrum underscores its strong discriminatory power in the predictive model. Increased serum concentrations of the myocardial injury biomarker cardiac troponin I (cTnI) and the sepsis-associated biomarker procalcitonin (PCT) both exhibited dose-dependent associations with elevated mortality risk in the model (**Figure 6**).

**Figure 7:**
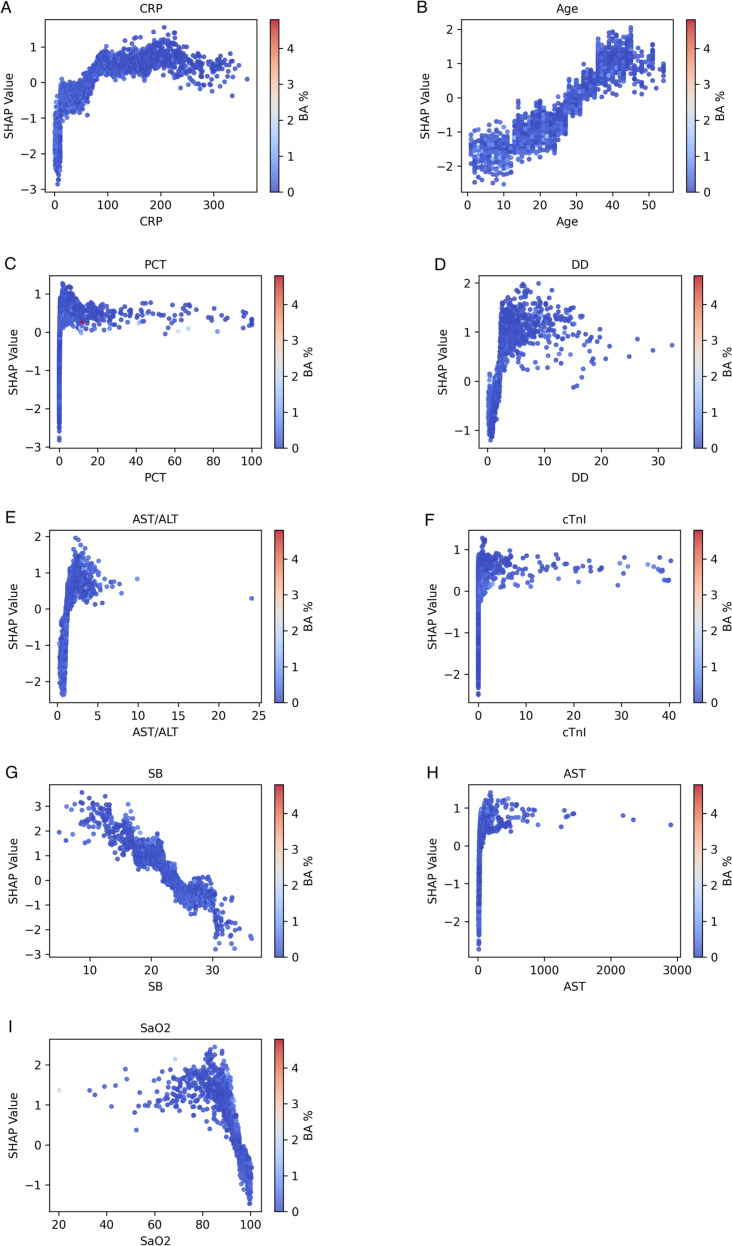
SHAP dependence plots [**(A)** CRP, **(B)** age, **(C)** PCT, **(D)** DD, **(E)** AST/ALT, **(F)** cTnI, **(G)** SB, **(H)** AST, **(I)** SaO_2_]. The x-axis represents the feature values, and the y-axis denotes the contribution of the feature to the prediction of COVID-19 mortality risk. Positive values indicate an increase in mortality risk, while negative values imply a decrease in mortality risk. The color gradient from blue to red represents the basophil percentage (BA%) from low to high.

In predicting COVID-19 mortality risk, biomarkers presented heterogeneous nonlinear associations with COVID-19 mortality risk in model analysis. CRP displayed a nonlinear biphasic statistical trend in the model. When CRP < 100 mg/L, most SHAP values were negative; within the high CRP range (approximately 100–300 mg/L), SHAP values gradually shifted toward positive values (**Figure 7A**). This indicated that lower CRP correlated with a stronger protective effect in reducing mortality risk, while higher CRP levels exert a more pronounced promoting effect on increasing mortality risk.​ ​Age showed a monotonically increasing association with COVID-19 mortality risk, implying that advancing age correlated with higher mortality risk (**Figure 7B**). Low PCT levels were associated with a statistically protective trend in COVID-19 mortality risk prediction (**Figure 7C**). DD levels exceeding 5 μg/mL tended to correspond to a rapid elevation in mortality risk (**Figure 7D**). AST/ALT ratio and AST both exhibited nonlinear association patterns: low values were primarily linked to a favorable prognostic trend, while high values were associated with an increased mortality risk (**Figure 7E**). Prognosis was relatively favorable when the AST < 200 U, but mortality risk rose sharply when the AST > 500 U (**Figure 7H**). For cTnI, the majority of data points clustered at the bottom of the SHAP plot, characterized by low BA%, near-zero SHAP values, and low cTnI levels. This suggested that in most COVID-19 patients, low cTnI levels had limited predictive contribution to mortality risk in the model. In contrast, the scattered points in the upper region—marked by positive SHAP values and elevated cTnI—represent a model-identified statistical high-risk signature. Elevated cTnI is significantly associated with increased mortality risk in COVID-19 (**Figure 7F**).​ With respect to SB, as its value increased, SHAP values gradually shifted from predominantly positive to negative, suggesting a complex nonlinear association with COVID-19 mortality risk in the model (**Figure 7G**).​ ​SaO_2_ showed a strong negative association with COVID-19 mortality risk in the model (**Figure 7I**). Notably, given the substantial missing proportions of PCT and SaO_2_, the generalizability of their observed feature importance is limited, and the relevant feature importance patterns should be interpreted in the context of the present study. Model results suggested that elevated levels of cTnI, CRP, AST/ALT ratio, AST, DD and advanced age were statistically associated with higher mortality risk. These indicators may exhibited interactive associations with BA% in the predictive model. As illustrated in **Figure 7G**, a high BA% strengthens the positive contribution of low SB, while low BA% reinforces the negative contribution of high SB.

#### 3.6.1 Model presentation

**Figure 8:**
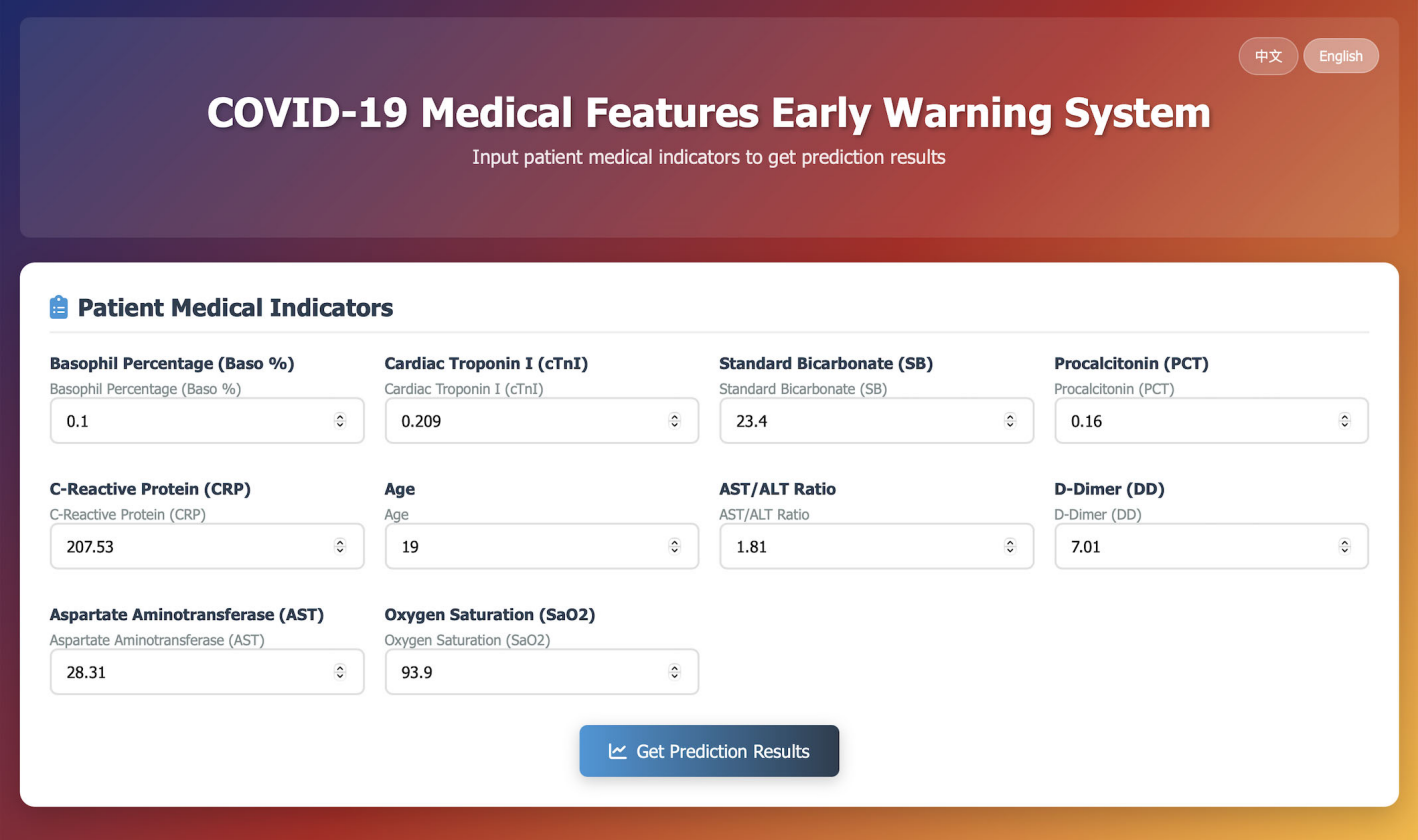
A web-based calculator for predicting mortality risk in older adults with COVID-19.

The finalized predictive model was deployed via an interactive web-based application. The platform of this model, provided in the **Supplementary Materials**, enables fellow researchers to replicate the analytical workflows and assess the validity of the model’s performance. A screenshot illustrating the generalized version of this model is presented in **Figure 8**.

## 4 Discussion

This study aimed to address the unmet clinical need for a targeted mortality risk prediction tool for older adults with COVID-19—a population proven to have the highest mortality risk yet underrepresented in existing machine learning (ML)-based prognostic models. By leveraging routine hematological indices and optimizing the LGBM algorithm through BO and SHAP-based feature selection, we developed a robust prognostic model with a mean AUC-ROC of 0.988 (post-BO) and maintained high performance (AUC-ROC: 0.960) even after reducing the features to the top 10.

Notably, the model addressed three critical limitations of previous COVID-19 prognostic research: (1) the overreliance on traditional linear scoring systems (e.g., CURB-65, PSI) that fail to capture complex multidimensional relationships (29, 30); (2) the tendency of most ML models to focus on the general population, with insufficient attention to older adult patients at high risk of severe illness (31); and (3) the black-box nature of many ML models, which hinders clinical trust. Through integrated analyses of PDPs and SHAP summary plots, this study not only validated the rationality of the model’s predictive logic but also uncovered novel pathological mechanisms linking routine biomarkers to COVID-19 mortality—thus providing a bridge between technical performance and clinical interpretability.

### 4.1 LGBM as the preferred model: performance and clinical applicability

Among the five ML algorithms compared (LR, SVM, RF, XGBoost, LGBM), LGBM emerged as the optimal model, with standout performance in key clinical metrics: a recall of 0.924 (minimizing false negatives), a Brier score of 0.064 (reducing prediction error), and a computational time 7.17% lower than XGBoost and approximately 88% lower than RF and SVM. These attributes are particularly critical for older adults with COVID-19, where missed identification of high-risk cases (false negatives) can lead to fatal delays in intervention, and rapid computation enables real-time risk stratification in busy clinical settings (e.g., emergency departments during surges). The BO-driven hyperparameter optimization further enhanced LGBM’s performance, increasing the mean AUC-ROC to 0.988—an improvement attributed to BO’s ability to systematically sample the hyperparameter space by probabilistic surrogate models, avoiding the inefficiency of grid and random search. This aligns with recent studies highlighting BO as a gold standard for ML model tuning in clinical prognostic tasks (32). Additionally, the 58.31% reduction in training time post-feature selection (retaining the top 10 SHAP-ranked features) did not compromise performance, confirming that the selected subset effectively captures core predictive information—which is pivotal for resource-limited settings where computational power may be constrained.

The LGBM model’s high negative predictive value (NPV: 0.923) and calibration accuracy (close alignment of predicted and observed outcomes along the diagonal of calibration curves) are of great clinical value. For older adult patients—many of whom present with non-specific symptoms (e.g., fatigue, anorexia) leading to delayed diagnosis (33)—a high NPV ensures that low-risk patients are not unnecessarily hospitalized, reducing the healthcare burden and iatrogenic risks (e.g., nosocomial infection). DCA further confirmed that LGBM and XGBoost outperformed other models in net clinical benefit across the threshold range of 0.2–0.8—which is clinically relevant, as thresholds below 0.2 typically correspond to low-risk patients (outpatient monitoring) and those above 0.8 to high-risk patients (ICU admission). The comparable net benefit of LGBM to XGBoost, combined with LGBM’s lower computational cost and greater stability, solidifies its suitability for long-term clinical deployment.

### 4.2 Model-derived patterns revealed by SHAP and PDP: associations between biomarkers and COVID-19 mortality risk

PDPs reflected nonlinear associations between core biomarkers and COVID-19 mortality risk, and identified statistical cut-off points for risk stratification in this cohort. In this cohort, CRP of 100 mg/L was a notable stratification cut-off. Below this level, mortality risk gradually increased alongside elevated inflammation; above 100 mg/L, mortality risk remained stably high, which may imply a potential adverse inflammatory cascade linking inflammation and organ injury (34). This statistical trend is consistent with the recognized pathogenic hypothesis of severe COVID-19. Older adults with weakened inflammatory regulation capacity may be more susceptible to excessive inflammatory responses. DD between 5–10 μg/mL represented another notable stratification boundary; higher DD values may reflect a higher likelihood of microcirculatory coagulation abnormalities. Exceeding this range was indicative of persistent microcirculatory thrombogenesis, a consequence of SARS-CoV-2-induced endothelial injury and inflammation-driven hypercoagulability (35). Given the high prevalence of age-related endothelial dysfunction in older adults, this threshold effectively identifies their elevated risk of fatal thrombotic complications (e.g., stroke, pulmonary embolism)—well-established major contributors to COVID-19 mortality. SaO_2_ of 85% was an obvious risk stratification cut-off in the model. SaO_2_ below 85% was closely correlated with higher mortality risk, which may be partly attributed to tissue hypoxia-related metabolic disorders. In older adult patients with diminished respiratory reserve, hypoxia rapidly impairs immune effector functions (e.g., neutrophil bactericidal activity), culminating in the collapse of inflammatory clearance mechanisms (36).

SHAP summary analysis indicated that BA% showed the strongest predictive weight in the model. Elevated BA% was correlated with positive SHAP values (higher mortality tendency), while low BA% corresponded to negative values (favorable prognostic correlation in statistics). This statistical phenomenon may be partially explained by the unique immune characteristics of older adults with COVID-19, age-related immunosenescence (e.g., impaired T cell function and macrophage phagocytosis) (37, 38)disrupted innate immune homeostasis, and BA%—as a niche yet critical mediator of antiviral immunity and inflammation (39)—served as a sentinel indicator for this imbalance.

Elevated BA% may potentially increase fatal risk via synergistic pathways, including excessive inflammatory response, immune imbalance and coagulation disorders. It triggered excessive release of proinflammatory mediators (histamine, leukotrienes, IL-4, IL-13), disrupting the alveolar-capillary barrier via MMP-9-mediated degradation of Claudin-5 and accelerating progression to acute respiratory distress syndrome (ARDS—the leading cause of death in severe cases) and facilitated cytokine storms through high-mobility group box 1 (HMGB1)-mediated basophil priming, ultimately inducing multiple organ dysfunction syndrome (MODS) (40–43). Strikingly, this inflammatory amplification effect was more pronounced in older adult patients, primarily attributed to a 51% reduction in AMPK signaling pathway activity in basophils of older adults compared with younger patients, which in turn enhances the regulatory sensitivity of BA% to IL-4/IL-13 (44). Additionally, elevated BA% impaired adaptive immunity by suppressing CD4+ T cell IFN-γ secretion and B cell IgG production, promoting persistent infection and disease progression (45). Overactivated basophils exhibited dysregulated heparin release: initial excessive release increased bleeding risk (e.g., pulmonary hemorrhage), while subsequent compensatory coagulation activation promoted microthrombosis (e.g., pulmonary embolism) (46). Elevated BA% further promotes reduced activity of plasma von Willebrand factor (vWF)-cleaving protease (ADAMTS13), resulting in the accumulation of ultra-large vWF multimers; concomitantly, the platelet aggregation rate increases, thereby forming a “immune activation-endothelial injury-thrombosis” triangular loop (47).Exacerbated by preexisting vascular endothelial injury in COVID-19 patients, this dysregulation further elevated lethal risks—a cohort study of 452 severe cases confirmed significantly higher coagulopathy rates and 28-day mortality in patients with elevated BA% (48).Consistent with prior studies, Palladino et al. (2021) reported that basophil dysfunction was closely associated with poor COVID-19 outcomes (49), and Li et al. (2020) observed a rebound increase in BA% during the severe disease phase, temporally coinciding with ARDS onset. Collectively, elevated BA% acts as a pivotal node connecting excessive inflammatory activation, immune impairment, and coagulopathy in COVID-19.

SHAP scatter plot further delineates the synergistic interplay between BA% and cTnI, wherein elevated BA% potentiates the prognostic utility of cTnI as a risk biomarker. This phenomenon underscores a pathological positive feedback loop of “immune dysregulation–myocardial injury”: heightened BA% denotes excessive activation of innate immunity (e.g., aberrant release of histamine and leukotrienes), which exacerbates myocardial cellular damage as reflected by elevated cTnI (50); reciprocally, damage-associated molecular patterns (DAMPs, such as HMGB1) released from injured myocardium further prime basophils, thereby amplifying proinflammatory cascades (43). Older adult patients, who often present with preexisting cardiac comorbidities, are particularly vulnerable to this deleterious loop, which also rationalizes the dominance of BA% as a pivotal predictive factor.

SHAP analysis further demonstrated that BA% regulates the effects of other biomarkers: high BA% exacerbated the risk-promoting effect of low SB, whereas low BA% potentiated the protective effect of high SB, indicating BA% functions as a potential “gatekeeper” for metabolic homeostasis. Mechanistically, this likely involves high BA% compromising renal compensatory mechanisms for acidosis via inflammation-mediated renal dysfunction, thereby shifting SB’s protective compensatory role to a risk-promoting signal (30). Clinically, this highlights the necessity of combined assessment of BA% and SB in managing acid-base balance in older adult patients, rather than exclusive dependence on SB as an isolated marker.

This study advances COVID-19 prognostic research across four key dimensions: First, unlike previous machine learning models focusing on the general population (31, 51), it specifically targets older adult patients—who account for 81% of COVID-19 deaths (U.S. CDC data (52))—with a high recall rate of 0.945 that aligns precisely with the clinical priority of identifying high-risk older adults. Second, in terms of feature practicality, whereas Smith et al.’s random forest model (AUC = 0.94) required 25 features (including chest CT, which has low penetration in primary care) (53), our study utilizes only 10 routine hematological indices, maintaining performance while reducing costs and radiation exposure—particularly critical for older adult patients with comorbidities. Third, for interpretability, unlike Zhang et al.’s support vector machine model (AUC = 0.92) that lacked mechanistic insights, it identifies intervention-guiding biomarkers (e.g., BA%>0.5%, AST>500 U) and validates the model via integrated SHAP and PDP analyses. Fourth, in model optimization, whereas Li et al.’s logistic regression model had a recall rate of only 0.72 due to unaddressed class imbalance (54), this study boosted the LGBM recall rate to 0.945 via RandomOverSampler and Bayesian optimization, minimizing false negatives in older adult patients.

## 5 Limitations

Limitations of the present study merit acknowledgment: First, missing data processing was a major methodological limitation of this study. Clinical laboratory indicators are selectively ordered based on clinical management demands, leading to inherent informative missingness. Although RF imputation effectively estimates missing values based on observed data patterns, it cannot fully represent the real clinical context behind unmeasured laboratory results and may introduce potential bias in the feature importance derived from the final model. Specifically, for variables with high missing proportions, such as PCT (35.31%) and SaO_2_ (28.29%), were largely attributable to clinical decision-making. Accordingly, the corresponding feature importance results should be interpreted with adequate caution to avoid over-interpretation. Second, this study utilized a retrospective single-center cohort (n=2393), which may compromise the model’s external validity. Additionally, only static baseline metrics at admission were included, with no incorporation of longitudinal in-hospital monitoring data—such dynamic data may better predict late-stage disease deterioration (55). Third, limitations also exist in clinical translation: the model lacks prospective validation and cost-effectiveness analysis. Further randomized controlled trials (RCTs) comparing clinical outcomes between “model-guided intervention” and “standard care” are needed to validate its actual efficacy in reducing mortality. Finally, mechanistic validation remains insufficient. Although SHAP/PDP analyses suggest potential pathological associations, the specific mechanisms regulating COVID-19 require further confirmation through biological studies.

Addressing these limitations, future work will focus on three key avenues: First, we will undertake multicenter prospective validation across 3–5 geographically diverse hospitals in China, enrolling patients across SARS-CoV-2 variants (e.g., JN.1) and age subgroups to systematically assess model generalizability. Second, we will develop a temporal prognostic model via integration of serial monitoring data (e.g., CRP, SaO_2_) and long short-term memory (LSTM) networks to capture disease progression trajectories with high precision. Third, facilitate clinical translation through development of an EHR-integrable web-based tool that delivers real-time risk scores and actionable clinical recommendations (e.g., BA>0.5% and cTnI>0.3 ng/mL: initiate anti-inflammatory therapy).

## 6 Conclusion

This study developed a TPE-LGBM model based on routine hematological indicators to predict mortality risk in older adult with COVID-19. The model demonstrated favorable predictive accuracy, computational efficiency, and interpretability, providing a potential reference for the early identification of high-risk older adult with COVID-19 in clinical practice. It also suggests the potential value of applying explainable machine learning to address unmet medical needs.

Prospective validation and clinical impact assessment were not performed in the present study; therefore, the model is not yet suitable for direct clinical implementation. Future studies incorporating multicenter validation and impact assessment are required to explore the model’s utility in risk stratification and potential rational allocation of medical resources.

## Data Availability

The original contributions presented in the study are included in the article/**Supplementary Material**. Further inquiries can be directed to the corresponding authors.
