# Supplementary material for: Explainable machine learning-based mortality risk stratification for older adults with COVID-19: pinpointing core immunological biomarkers and revealing dose-threshold effects
Source: Front Immunol. 2026 May 25;17:1789048. doi: 10.3389/fimmu.2026.1789048 (PMC13243428; doi:10.3389/fimmu.2026.1789048)
Supplement: Supplementary file 1 [file Table1.docx]

**Table S1. Baseline Characterstics of the Study Cohort Stratified by Survival Status**

| **V****ariables** | **Category** | **Overall** | **Non-survivors** | **Survivors** | **Missing（n）** | **Missin（%）** |
| --- | --- | --- | --- | --- | --- | --- |
| **ABO Typing** | A | 146 (26.40%) | 34 (31.19%) | 112 (25.23%) | 1840 | 76.89 |
|  | B | 191 (34.54%) | 37 (33.94%) | 154 (34.68%) |  |  |
|  | O | 166 (30.02%) | 31 (28.44%) | 135 (30.41%) |  |  |
|  | AB | 50 (9.04%) | 7 (6.42%) | 43 (9.68%) |  |  |
| **Gender** | Male | 1397(58.38%) | 252 (67.74%) | 1145 (56.66%) | 0 | 0.00 |
|  | Female | 996 (41.62%) | 120 (32.26%) | 876 (43.34%) |  |  |
| **Smoking History** | Smoking | 480 (22.61%) | 71 (21.78%) | 409 (22.76%) | 270 | 11.28 |
|  | Non-smoking | 1643(77.39%) | 255 (78.22%) | 1388 (77.24%) |  |  |
| **Drinking History** | Drinking | 336 (15.85%) | 54 (16.51%) | 282 (15.73%) | 273 | 11.41 |
|  | Non-drinking | 1784(84.15%) | 273 (83.49%) | 1511 (84.27%) |  |  |
| **Age (year)** | [50-60) | 295(12.33%) | 22 (5.91%) | 273 (13.51%) | 0 | 0.00 |
|  | [60-70) | 554(23.15%) | 48 (12.90%) | 506 (25.04%) |  |  |
|  | [70-80) | 631(26.37%) | 90 (24.19%) | 541 (26.77%) |  |  |
|  | [80-90) | 715(29.88%) | 154 (41.40%) | 561 (27.76%) |  |  |
|  | [90-100) | 193 (8.07%) | 56 (15.05%) | 137 (6.78%) |  |  |
|  | [100-110] | 5 (0.21%) | 2 (0.54%) | 3 (0.15%) |  |  |
| **pH Urine** | [5-5.5) | 356(20.60%) | 88 (38.94%) | 268 (17.84%) | 665 | 27.79 |
|  | [5.5-6) | 100 (5.79%) | 21 (9.29%) | 79 (5.26%) |  |  |
|  | [6-6.5) | 755(43.69%) | 87 (38.50%) | 668 (44.47%) |  |  |
|  | [6.5-7) | 284(16.44%) | 16 (7.08%) | 268 (17.84%) |  |  |
|  | [7-7.5) | 168 (9.72%) | 9 (3.98%) | 159 (10.59%) |  |  |
|  | [7.5-8) | 45 (2.60%) | 3 (1.33%) | 42 (2.80%) |  |  |
|  | [8-8.5) | 14 (0.81%) | 1 (0.44%) | 13 (0.87%) |  |  |
|  | [8.5-9] | 6 (0.35%) | 1 (0.44%) | 5 (0.33%) |  |  |
| **SAA (mg/L)** | | 245 | 263.69 (102.43) | 166.58(128.36) | 2148 | 89.76 |
| **TBIL (µmol/L)** | | 2259 | 15.82 (18.70) | 13.97 (16.10) | 134 | 5.60 |
| **PA(mg/L)** | | 1956 | 102.58 (59.58) | 161.47 (75.81) | 437 | 18.26 |
| **TC(mmol/L)** | | 1594 | 3.89 (1.32) | 4.18 (1.25) | 799 | 33.39 |
| **IBIL(µmol/L)** | | 1956 | 7.10 (5.61) | 7.76 (6.23) | 437 | 18.26 |
| **ALB(g/L)** | | 2261 | 29.74 (5.00) | 33.47 (5.37) | 132 | 5.52 |
| **ALP (U/L)** | | 2260 | 90.07 (50.46) | 88.58 (55.33) | 133 | 5.56 |
| **AST/ALT** | | 2260 | 1.83 (0.93) | 1.38 (0.94) | 133 | 5.56 |
| **TP(g/L)** | | 2260 | 61.46 (8.62) | 63.23 (7.81) | 133 | 5.56 |
| **GLU (mmol/L)** | | 2054 | 9.77 (5.70) | 7.99 (4.15) | 339 | 14.17 |
| **Amy(U/L)** | | 296 | 130.08 (228.44) | 90.99 (174.00) | 2097 | 87.63 |
| **TG(mmol/L)** | | 1594 | 1.46 (0.77) | 1.38 (0.75) | 799 | 33.39 |
| **α-HBDH(U/L)** | | 644 | 313.09 (336.13) | 216.33(315.21) | 1749 | 73.09 |
| **cTnI (ug/L)** | | 2013 | 1.36 (5.23) | 0.40 (2.28) | 380 | 15.88 |
| **TBA(µmol/L)** | | 1956 | 6.33 (14.66) | 6.32 (18.27) | 437 | 18.26 |
| **ALB/GLO** | | 1956 | 1.01 (0.29) | 1.22 (0.35) | 437 | 18.26 |
| **CK-MB(ug/L)** | | 2037 | 9.38 (46.03) | 5.04 (10.31) | 356 | 14.88 |
| **LPS(U/L)** | | 280 | 219.62 (469.50) | 178.39(545.07) | 2113 | 88.30 |
| **LDL-C(mmol/L)** | | 1594 | 2.28 (1.05) | 2.54 (1.06) | 799 | 33.39 |
| **PCT (ng/ml)** | | 1548 | 6.45 (18.73) | 1.73 (8.53) | 845 | 35.31 |
| **ALT (U/L)** | | 2260 | 45.13 (95.20) | 33.63 (62.49) | 133 | 5.56 |
| **hs-CRP(mg/L)** | | 241 | 98.20 (84.97) | 60.60 (60.98) | 2152 | 89.93 |
| **LDH(U/L)** | | 1982 | 437.54 (398.40) | 271.72(270.22) | 411 | 17.18 |
| **CRP (mg/L)** | | 1896 | 117.63 (83.62) | 62.45 (63.77) | 497 | 20.77 |
| **BNP(pg/ml)** | | 1555 | 452.61 (781.07) | 261.12(585.04) | 838 | 35.02 |
| **P (mmol/L)** | | 1938 | 1.14 (0.55) | 1.06 (0.35) | 455 | 19.01 |
| **UA(µmol/L)** | | 2237 | 402.21 (221.71) | 305.80(138.60) | 156 | 6.52 |
| **Cys-C (mg/L)** | | 1620 | 2.63 (2.18) | 1.92 (2.01) | 773 | 32.30 |
| **CREA (mmol/L)** | | 2237 | 169.54 (190.02) | 116.04(176.51) | 156 | 6.52 |
| **apoAI(mmol/L)** | | 1594 | 1.02 (0.26) | 1.19 (0.31) | 799 | 33.39 |
| **GGT (U/L)** | | 2258 | 53.28 (64.56) | 55.26 (92.35) | 135 | 5.64 |
| **CO2((mmol/L))** | | 1912 | 22.32 (4.91) | 24.84 (3.66) | 481 | 20.10 |
| **HDL-C(mmol/L)** | | 1594 | 0.94 (0.30) | 1.00 (0.31) | 799 | 33.39 |
| **CK(U/L)** | | 650 | 693.10 (1825.89) | 141.22(474.31) | 1743 | 72.84 |
| **DBIL(µmol/L)** | | 1956 | 8.58 (15.31) | 6.20 (11.66) | 437 | 18.26 |
| **AST (U/L)** | | 2271 | 82.69 (229.26) | 39.15 (92.34) | 122 | 5.10 |
| **eGFR** | | 784 | 45.46 (25.11) | 56.88 (27.53) | 1609 | 67.24 |
| **apoB(mmol/L)** | | 1594 | 0.75 (0.28) | 0.77 (0.24) | 799 | 33.39 |
| **Ca(mmol/L)** | | 2309 | 2.01 (0.20) | 2.09 (0.18) | 84 | 3.51 |
| **NT-ProBNP(pg/ml)** | | 395 | 6887.08 (9614.69) | 3092.01(5221.77) | 1998 | 83.49 |
| **GLO(g/L)** | | 1956 | 30.27 (7.02) | 28.21 (5.31) | 437 | 18.26 |
| **UREA (mmol/L)** | | 2236 | 14.97 (11.37) | 8.20 (6.87) | 157 | 6.56 |
| **HBsAg（COI）** | | 1808 | 24.69 (197.11) | 34.54 (258.99) | 585 | 24.45 |
| **CA153(KU/L）** | | 832 | 12.29 (16.13) | 11.59 (31.31) | 1561 | 65.23 |
| **AFP(ug/L)** | | 755 | 6.38 (28.72) | 9.89 (172.41) | 1638 | 68.45 |
| **NSE(ug/L)** | | 999 | 9.86 (10.42) | 7.42 (11.52) | 1394 | 58.25 |
| **F-PSA(ug/L)** | | 492 | 1.13 (2.33) | 0.58 (1.43) | 1901 | 79.44 |
| **CEA(ug/L)** | | 991 | 5.05 (8.86) | 12.50 (205.09) | 1402 | 58.59 |
| **CYFRA211(ug/L)** | | 266 | 18.04 (37.10) | 5.76 (10.87) | 2127 | 88.88 |
| **CA199(KU/L）** | | 748 | 23.20 (28.24) | 79.16 (1352.48) | 1645 | 68.74 |
| **CA125(KU/L）** | | 891 | 43.06 (160.06) | 30.43 (204.29) | 1502 | 62.77 |
| **CA242(KU/L）** | | 739 | 4.59 (7.23) | 4.70 (11.02) | 1654 | 69.12 |
| **T-PSA(ug/L)** | | 692 | 5.94 (13.57) | 2.93 (7.25) | 1701 | 71.08 |
| **PaCO2(mmHg)** | | 1719 | 37.02 (13.99) | 37.22 (6.76) | 674 | 28.17 |
| **P50(mmHg)** | | 1707 | 27.31 (3.86) | 26.00 (2.57) | 686 | 28.67 |
| **SaO2 (%)** | | 1716 | 91.76 (8.89) | 94.97 (6.64) | 677 | 28.29 |
| **ABE(mmol/L)** | | 1717 | -3.00 (6.00) | 0.11 (4.00) | 676 | 28.25 |
| **RI(%)** | | 1573 | 109.63 (156.31) | 58.78 (75.79) | 820 | 34.27 |
| **PO2（A-a）（mmHg)** | | 1502 | 68.53 (83.97) | 42.17 (48.52) | 891 | 37.23 |
| **PH(T)(℃)** | | 1717 | 7.38 (0.11) | 7.42 (0.06) | 676 | 28.25 |
| **SB(mmol/L)** | | 1716 | 22.06 (4.64) | 24.58 (3.28) | 677 | 28.29 |
| **PaO2（T）(mmHg)** | | 1715 | 85.31 (45.47) | 93.59 (40.54) | 678 | 28.33 |
| **ctCO2（B）(mmol/L)** | | 1715 | 23.15 (10.42) | 25.76 (11.11) | 678 | 28.33 |
| **SBE(mmol/L)** | | 1719 | -3.36 (6.41) | -0.05 (4.43) | 674 | 28.17 |
| **PaO2 (mmHg)** | | 1718 | 85.43 (45.47) | 93.75 (40.68) | 675 | 28.21 |
| **PaCO2(T)(mmHg)** | | 1717 | 36.97 (13.98) | 37.18 (6.71) | 676 | 28.25 |
| **FiO2(%)** | | 1719 | 25.01 (13.36) | 22.86 (8.04) | 674 | 28.17 |
| **ctCO2（P）(mmol/L)** | | 1717 | 26.65 (12.01) | 29.90 (12.76) | 676 | 28.25 |
| **EO #(×10^9^/L)** | | 2299 | 0.02 (0.07) | 0.06 (0.12) | 94 | 3.93 |
| **PCT(%)** | | 2296 | 0.19 (0.09) | 0.20 (0.09) | 97 | 4.05 |
| **MO #(×10^9^/L)** | | 2297 | 0.48 (0.71) | 0.50 (0.92) | 96 | 4.01 |
| **MCHC(g/L)** | | 2299 | 333.50 (12.65) | 335.69 (12.05) | 94 | 3.93 |
| **RDW (fl)** | | 2299 | 13.96 (1.88) | 13.63 (2.01) | 94 | 3.93 |
| **RBC(×10^12^/L)** | | 2299 | 3.88 (0.87) | 3.91 (0.82) | 94 | 3.93 |
| **MCH(pg)** | | 2299 | 30.56 (2.92) | 30.72 (2.50) | 94 | 3.93 |
| **Hb (g/L)** | | 1161 | 120.09 (21.48) | 123.28 (19.97) | 1232 | 51.48 |
| **LY %(%)** | | 2297 | 10.58 (10.34) | 18.90 (12.31) | 96 | 4.01 |
| **EO %(%)** | | 2299 | 0.28 (0.79) | 0.91 (1.58) | 94 | 3.93 |
| **BA #(×10^9^/L)** | | 2299 | 0.01 (0.01) | 0.02 (0.24) | 94 | 3.93 |
| **P-LCR(%)** | | 2296 | 29.02 (9.10) | 26.35 (8.37) | 97 | 4.05 |
| **RDW(%)** | | 2299 | 46.35 (6.34) | 45.10 (6.38) | 94 | 3.93 |
| **HCT(%)** | | 2299 | 35.53 (8.03) | 35.63 (7.36) | 94 | 3.93 |
| **LY #(×10^9^/L)** | | 2253 | 0.70 (0.56) | 1.16 (2.02) | 140 | 5.85 |
| **WBC (× 10^9^/L)** | | 2299 | 9.44 (7.34) | 7.42 (14.91) | 94 | 3.93 |
| **PDW (fl)** | | 2296 | 15.93 (1.47) | 15.70 (1.79) | 97 | 4.05 |
| **MPV(fl)** | | 2296 | 10.38 (1.32) | 10.01 (1.22) | 97 | 4.05 |
| **ESR(mm/h)** | | 886 | 45.21 (28.87) | 36.69 (22.82) | 1507 | 62.98 |
| **NE #(×10^9^/L)** | | 2299 | 8.14 (6.53) | 5.15 (4.00) | 94 | 3.93 |
| **NE %(%)** | | 2299 | 83.41 (12.59) | 72.18 (14.27) | 94 | 3.93 |
| **MO %(%)** | | 2297 | 5.60 (3.91) | 7.65 (4.23) | 96 | 4.01 |
| **PLT (× 10^9^/L)** | | 2299 | 186.34 (94.53) | 203.72 (96.04) | 94 | 3.93 |
| **BA %(%)** | | 2299 | 0.12 (0.20) | 0.25 (0.26) | 94 | 3.93 |
| **MCV(fl)** | | 2299 | 91.61 (7.82) | 91.46 (6.25) | 94 | 3.93 |
| **RBC Urine（/uL）** | | 1591 | 1804.40 (10129.69) | 265.59 (2897.24) | 802 | 33.51 |
| **BACT Urine（/uL）** | | 1452 | 611.04 (999.96) | 333.34 (745.93) | 941 | 39.32 |
| **WBC Urine（/HP）** | | 1451 | 22.58 (82.51) | 42.49 (586.49) | 942 | 39.36 |
| **SG Urine** | | 1719 | 1.02 (0.01) | 1.02 (0.01) | 674 | 28.17 |
| **WBC Urine（/uL）** | | 1531 | 26.29 (56.14) | 19.54 (49.14) | 862 | 36.02 |
| **MUCS Urine（/uL）** | | 1580 | 81.86 (153.77) | 172.31 (285.16) | 813 | 33.97 |
| **TT (s)** | | 1921 | 20.17 (8.31) | 18.45 (3.90) | 472 | 19.72 |
| **PT%（%）** | | 1924 | 82.34 (18.72) | 93.68 (17.02) | 469 | 19.60 |
| **ATIII（%）** | | 728 | 74.69 (17.73) | 83.46 (17.83) | 1665 | 69.58 |
| **INR** | | 1924 | 1.21 (0.65) | 1.07 (0.26) | 469 | 19.60 |
| **PT-R** | | 1924 | 1.16 (0.56) | 1.05 (0.18) | 469 | 19.60 |
| **PT (s)** | | 1924 | 15.36 (6.53) | 13.87 (2.40) | 469 | 19.60 |
| **DD (ug/mL)** | | 1955 | 3.26 (4.14) | 1.55 (2.40) | 438 | 18.30 |
| **Fbg (g/L)** | | 1923 | 5.34 (2.00) | 4.88 (1.66) | 470 | 19.64 |
| **APTT (s)** | | 1923 | 42.16 (16.71) | 37.43 (7.14) | 470 | 19.64 |
| **FDP (ug/mL)** | | 724 | 20.63 (27.10) | 10.08 (17.23) | 1669 | 69.75 |
| **FT3(pmol/L)** | | 818 | 2.56 (0.92) | 3.49 (0.98) | 1575 | 65.82 |
| **FT4(pmol/L)** | | 857 | 13.96 (2.89) | 15.09 (2.89) | 1536 | 64.19 |
| **TPOAb（U/mL）** | | 315 | 57.80 (51.41) | 71.65 (117.22) | 2078 | 86.84 |
| **TSH(mIU/L)** | | 864 | 1.62 (3.54) | 2.08 (5.86) | 1529 | 63.89 |
| **GH(ng/mL)** | | 360 | 1.39 (1.44) | 0.90 (0.96) | 2033 | 84.96 |
| **T-SPOT** | | 314 | 201.90 (190.40) | 192.19 (186.24) | 2079 | 86.88 |
| **Na+ (mmol/L)** | | 2329 | 138.71 (9.20) | 137.41 (5.99) | 64 | 2.67 |
| **Mg (mmol/L)** | | 1912 | 0.87 (0.15) | 0.85 (0.12) | 481 | 20.10 |
| **Ca2+(mmol/L)** | | 1684 | 0.98 (0.12) | 0.99 (0.10) | 709 | 29.63 |
| **K+ (mmol/L)** | | 2329 | 4.03 (0.82) | 3.85 (0.58) | 64 | 2.67 |
| **Cl- (mmol/L)** | | 2329 | 104.02 (8.69) | 102.89 (6.36) | 64 | 2.67 |
| **HbA1c（%）** | | 912 | 7.31 (2.31) | 7.07 (1.85) | 1481 | 61.89 |

Note: SAA: Serum Amyloid A; TBIL: Total Bilirubin; PA: Prealbumin; TC: Total Cholesterol; IBIL: Indirect Bilirubin; ALB: Albumin; ALP: Alkaline Phosphatase; AST/ALT: Aspartate Aminotransferase/Alanine Aminotransferase Ratio; TP: Total Protein; GLU: Glucose; Amy: Amylase; TG: Triglyceride; α-HBDH: α-Hydroxybutyric Dehydrogenase; cTnI: Cardiac Troponin I; TBA: Total Bile Acid; ALB/GLO: Albumin/Globulin Ratio; CK-MB: Creatine Kinase-MB; LPS: Lipopolysaccharide; LDL-C: Low-Density Lipoprotein Cholesterol; PCT: Procalcitonin; ALT: Alanine Aminotransferase; hs-CRP: High-Sensitivity C-Reactive Protein; LDH: Lactate Dehydrogenase; CRP: C-Reactive Protein; BNP: Brain Natriuretic Peptide; P: Phosphorus; UA: Uric Acid; Cys-C: Cystatin C; CREA: Creatinine; apoAI: Apolipoprotein AI; GGT: Gamma-Glutamyl Transferase; CO2: Carbon Dioxide; HDL-C: High-Density Lipoprotein Cholesterol; CK: Creatine Kinase; DBIL: Direct Bilirubin; AST: Aspartate Aminotransferase; eGFR: Estimated Glomerular Filtration Rate; apoB: Apolipoprotein B; Ca: Calcium; NT-ProBNP: N-Terminal Pro-Brain Natriuretic Peptide; GLO: Globulin; UREA: Urea; HBsAg: Hepatitis B Surface Antigen; CA153: Cancer Antigen 15-3; AFP: Alpha-Fetoprotein; NSE: Neuron-Specific Enolase; F-PSA: Free Prostate-Specific Antigen; CEA: Carcinoembryonic Antigen; CYFRA21-1: Cytokeratin 19 Fragment 21-1; CA199: Cancer Antigen 19-9; CA125: Cancer Antigen 125; CA242: Cancer Antigen 242; T-PSA: Total Prostate-Specific Antigen; PaCO2: Partial Pressure of Arterial Carbon Dioxide; P50: Partial Pressure of Oxygen at 50% Hemoglobin Saturation; SaO2: Arterial Oxygen Saturation; ABE: Actual Base Excess; RI: Resistance Index; PO2 (A-a): Alveolar-Arterial Oxygen Partial Pressure Difference; PH: Temperature-Corrected pH; SB: Standard Bicarbonate; PaO2 (T): Temperature-Corrected Partial Pressure of Arterial Oxygen; ctCO2 (B): Calculated Total Carbon Dioxide (Blood); SBE: Standard Base Excess; PaO2: Partial Pressure of Arterial Oxygen; PaCO2(T): Temperature-Corrected Partial Pressure of Arterial Carbon Dioxide; FiO2: Fraction of Inspired Oxygen; ctCO2 (P): Calculated Total Carbon Dioxide (Plasma); EO #: Eosinophil Count; PCT(%): Plateletcrit; MO#: Monocyte Count; MCHC: Mean Corpuscular Hemoglobin Concentration; RDW: Red Cell Distribution Width (femtoliter); RBC: Red Blood Cell Count; MCH: Mean Corpuscular Hemoglobin; Hb: Hemoglobin; LY%: Lymphocyte Percentage; EO%: Eosinophil Percentage; BA#: Basophil Count; P-LCR: Platelet Large Cell Ratio; RDW(%): Red Cell Distribution Width (Percentage); HCT: Hematocrit; LY #: Lymphocyte Count; WBC: White Blood Cell Count; PDW: Platelet Distribution Width; MPV: Mean Platelet Volume; ESR: Erythrocyte Sedimentation Rate; NE#: Neutrophil Count; NE%: Neutrophil Percentage; MO%: Monocyte Percentage; PLT: Platelet Count; BA%: Basophil Percentage; MCV: Mean Corpuscular Volume; RBC Urine: Red Blood Cell Count in Urine; BACT Urine: Bacteria Count in Urine; WBC Urine: White Blood Cell Count in Urine per High-Power Field; SG Urine: Specific Gravity of Urine; WBC Urine: White Blood Cell Count in Urine per Microliter; MUCS Urine: Mucus in Urine; TT: Thrombin Time; PT%: Prothrombin Time Percentage; ATIII: Antithrombin III; INR: International Normalized Ratio; PT-R: Prothrombin Time Ratio; PT: Prothrombin Time; DD: D-Dimer; Fbg: Fibrinogen; APTT: Activated Partial Thromboplastin Time; FDP: Fibrin Degradation Products; FT3: Free Triiodothyronine; FT4: Free Thyroxine; TPOAb: Thyroid Peroxidase Antibody; TSH: Thyroid-Stimulating Hormone; GH: Growth Hormone; T-SPOT: T-SPOT.TB Test; Na+: Sodium; Mg: Magnesium; Ca2+: Ionized Calcium; K+: Potassium; Cl-: Chloride; HbA1c: Glycated Hemoglobin.
