# Supplementary material for: Explainable machine learning-based mortality risk stratification for older adults with COVID-19: pinpointing core immunological biomarkers and revealing dose-threshold effects
Source: Front Immunol. 2026 May 25;17:1789048. doi: 10.3389/fimmu.2026.1789048 (PMC13243428; doi:10.3389/fimmu.2026.1789048)
Supplement: Supplementary file 2 [file Presentation1.zip › Supplementary Material 2,platform/COVID-19 Medical Features Early Warning System.html]

COVID-19 Medical Features Early Warning System / COVID-19医学特征预警系统


中文
English

# COVID-19医学特征预警系统

输入患者医学指标，获取预测结果

## 患者医学指标

嗜碱性粒细胞百分比 (Baso %)

Basophil Percentage (Baso %)

心肌肌钙蛋白I (cTnI)

Cardiac Troponin I (cTnI)

标准碳酸氢盐 (SB)

Standard Bicarbonate (SB)

降钙素原 (PCT)

Procalcitonin (PCT)

C反应蛋白 (CRP)

C-Reactive Protein (CRP)

年龄

Age

AST/ALT比值

AST/ALT Ratio

D-二聚体 (DD)

D-Dimer (DD)

天门冬氨酸氨基转移酶 (AST)

Aspartate Aminotransferase (AST)

氧饱和度 (SaO2)

Oxygen Saturation (SaO2)

获取预测结果

正在分析数据，请稍候...

### 预测结果

医学特征预测系统 © 2023 | 仅供医疗专业人员参考
